# Biobased Poly(itaconic Acid-*co*-10-Hydroxyhexylitaconic Acid)s: Synthesis and Thermal Characterization

**DOI:** 10.3390/ma13122707

**Published:** 2020-06-14

**Authors:** Yuji Aso, Mei Sano, Ryoki Yada, Tomonari Tanaka, Takashi Aoki, Hitomi Ohara, Takahiro Kusukawa, Keiji Matsumoto, Kazuhito Wada

**Affiliations:** 1 Department of Biobased Materials Science, Kyoto Institute of Technology, 1 Hashigami-cho, Matsugasaki, Sakyo-ku, Kyoto 606-8585, Japan; d6861001@edu.kit.ac.jp (M.S.); m7661008@edu.kit.ac.jp (R.Y.); t-tanaka@kit.ac.jp (T.T.); t-aoki@kit.ac.jp (T.A.); ohara@kit.ac.jp (H.O.); 2 Department of Chemistry and Materials Technology, Kyoto Institute of Technology, 1 Hashigami-cho, Matsugasaki, Sakyo-ku, Kyoto 606-8585, Japan; kusu@kit.ac.jp; 3 Corporate Research & Business Division, Kaneka Corporation, 2-3-18 Nakanoshima, Kita-ku, Osaka 530-8288, Japan; Keiji.Matsumoto@kaneka.co.jp (K.M.); Kazuhito.Wada@kaneka.co.jp (K.W.)

**Keywords:** itaconic acid, 10-hydroxyhexylitaconic acid, radical polymerization, renewable polymer

## Abstract

Renewable vinyl compounds itaconic acid (IA) and its derivative 10-hydroxyhexylitaconic acid (10-HHIA) are naturally produced by fungi from biomass. This provides the opportunity to develop new biobased polyvinyls from IA and 10-HHIA monomers. In this study, we copolymerized these monomers at different ratios through free radical aqueous polymerization with potassium peroxodisulfate as an initiator, resulting in poly(IA-*co*-10-HHIA)s with different monomer compositions. We characterized the thermal properties of the polymers by thermogravimetric analysis (TGA) and Fourier-transform infrared spectroscopy (FT-IR). The nuclear magnetic resonance analysis and the gel permeation chromatography showed that the polymerization conversion, yield, and the molecular weights (weight-averaged *M*w and number-averaged *M*n) of the synthesized poly(IA-*co*-10-HHIA)s decreased with increasing 10-HHIA content. It is suggested that the hydroxyhexyl group of 10-HHIA inhibited the polymerization. The TGA results indicated that the poly(IA-*co*-10-HHIA)s continuously decomposed as temperature increased. The FT-IR analysis suggested that the formation of the hydrogen bonds between the carboxyl groups of IA and 10-HHIA in the polymer chains was promoted by heating and consequently the polymer dehydration occurred. To the best of our knowledge, this is the first time that biobased polyvinyls were synthesized using naturally occurring IA derivatives.

## 1. Introduction

Owing to the growing environmental interest, the development of renewable polymer materials derived from biomass is increasing in popularity [1,2,3,4]. To develop polymers with unique properties, novel monomer type building blocks are in demand because the monomer unit predominantly affects the polymer properties [5,6,7]. Some renewable monomers that are produced by microbes from biomass consist of larger carbon numbers (higher than C5) [8]. These monomers are barely synthesized from petroleum resources and possess unique structural characteristics increasing their potential to be applied as new monomers.

Itaconic acid (IA) is one of the most promising renewable vinyl compounds commercially produced using fungi, mostly *Aspergillus terreus* [9,10,11,12]. IA is a sustainable monomer because it is fermentatively produced from biomass. To date, many IA copolymers have been synthesized from IA by free radical copolymerization (e.g., with methyl methacrylate (MMA)) or polycondensation (e.g., with diols) [8,13,14,15,16,17,18,19,20]. For instance, Ranjha et al., have synthesized poly(MMA-*co*-IA) by free radical copolymerization using crosslinkers for development of controlled drug delivery [19]. On the other hand, Dai et al., have synthesized a series of polyesters by melt polycondensation of IA with diols and glycerol [20]. The resulting copolymers further copolymerized with acrylated epoxidized soybean oil showed great potential for applications as coatings, adhesives, and composites. Specifically, some papers reported that IA-based polyvinyls have cation-exchange properties [21,22,23]. Interestingly, naturally occurring IA derivatives possessing a vinyl group have been found as metabolic products from fungi and lichens. This suggests that it is possible to synthesize new biobased polymers from these IA derivatives. However, there is no report on the polymers synthesized using naturally occurring IA derivatives.

Recently, we have developed a new screening method for microbes from soil to produce IA [24,25,26]. In our screening study, we have also isolated a fungus *Aspergillus niger* S17-5 producing two IA derivatives from glucose, 9-hydroxyhexylitaconic acid (9-HHIA) and 10-hydroxyhexylitaconic acid (10-HHIA) [27]. These compounds possess an alkyl chain with a hydroxy group. Therefore, it might be possible to synthesize novel biobased polyvinyls excellent in impact resistance, tensile strength, and moldability, compared to IA homopolymers. The production titer of 10-HHIA is higher than that of 9-HHIA; therefore, 10-HHIA would be the preferred monomer for the polymer synthesis.

**Figure 1:**
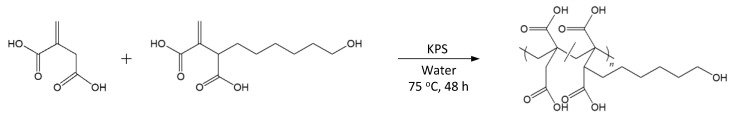
Synthesis of poly(IA-*co*-10-HHIA)s.

The present study reports on the free radical polymerization of poly(IA-*co*-10-HHIA)s with IA and 10-HHIA monomers in water using potassium peroxodisulfate (KPS) as initiator (Figure 1). The thermal properties of the polymers were characterized with thermogravimetric analysis (TGA) and Fourier-transform infrared spectroscopy (FT-IR).

## 2. Materials and Methods

### 2.1. Materials

IA (Molecular weight: 130.1) was purchased from Wako Pure Chemical Corp. (Osaka, Japan). KPS, sodium dihydrogenphosphate, disodium hydrogenphosphate, deuterium oxide, and sodium hydroxide were purchased from Nacalai Tesque Inc. (Kyoto, Japan). All reagents were used without further purification.

### 2.2. Preparation of 10-HHIA

The 10-HHIA (Molecular weight: 230.2 g mol−^1^) was obtained from the culture supernatant of *A. niger* S17-5 according to a method described elsewhere [27]. Briefly, *A. niger* S17-5 was cultured in 1 L of a GM2 liquid medium (per 1 L: 130 g glycerol, 0.154 g MgSO_4_·7H_2_O, 0.19 mg FeCl_2_·4H_2_O, 0.46 g NH_4_NO_3_, 15.4 mg KH_2_PO_4_, 96 mg CaCl_2_, 1.2 mg ZnSO_4_·7H_2_O, 2.3 mg CuSO_4_·5H_2_O) [28]. The culture was centrifuged to obtain the supernatant. The supernatant typically contained 10-HHIA at a concentration of 0.5 g L^−1^. The supernatant was purified using a preparative high-performance liquid chromatograph (HPLC, LaChrom Elite, Hitachi High-Technologies, Tokyo, Japan) equipped with a preparative HPLC Inertsil ODS 10 μm column (GL sciences, Tokyo, Japan). The 10-HHIA was eluted using a water/acetonitrile/trifluoro acetic acid solution (flow rate: 5 mL min^−1^). The eluate was monitored at an absorbance of 210 nm. The eluate was dried by freeze dehydration, resulting in the purified 10-HHIA (approx. 0.1 g).

### 2.3. Copolymerization

Different IA/10-HHIA monomer ratios were used (200/0, 160/40, 100/100, and 0/200 μmol in feed). The corresponding monomer ratio and 2 μmol KPS were added to 0.4 mL of water in a glass ampule. After the mixtures were degassed three times by freeze-thaw, the glass ampule was sealed and then placed in an oil bath at 75 °C for 48 h. Then, the reaction mixtures were dialyzed in water at 25 °C for 3 days with a Spectra/Por 7 dialysis membrane (Molecular weight cut-off: 1 kDa) (Spectrum Laboratories Inc., Rancho Dominguez, CA, USA). The dialyzed solutions were dried by freeze dehydration, resulting in the purified poly(IA-*co*-10-HHIA)s.

### 2.4. Measurements

The ^1^H NMR spectra were recorded on a Bruker AV-300 (Billerica, MA, USA). All samples were analyzed by ^1^H NMR with D_2_O after freeze dehydration. Especially, when samples of poly(IA-*co*-10-HHIA) with IA/10-HHIA monomer ratios of 100/100 and 0/200 were analyzed by NMR, an aliquot of 10 mM NaOH in D_2_O was used as a solvent to completely dissolve the synthesized polymers. The conversion of the synthesized copolymers was calculated as follows:

Conversion (%) = (1 – A_m_/(A_m_ + A_p_)) × 100

A_m_: the area of vinylidene proton signals at 6.5 ppm;

A_p_: the area of copolymer proton signals.

The gel permeation chromatography (GPC) was conducted with a PU-2089 pump (JASCO, Tokyo, Japan), a CO-2065 column oven (JASCO), and an RI-2031 refractive index detector (JASCO). A Shodex OHpak SB-804 HQ (8.0 × 300 mm, Showa Denko K.K., Tokyo, Japan) column was used with 20 mM phosphate buffer (pH 7.0) as the eluent (flow rate: 0.5 mL min^−1^ at 30 °C). The molecular weights were calibrated against pullulan standards according to the method for GPC analysis of IA-derived polymers [16].

The TGA was carried out with a Discovery TGA (TA instruments, New Castle, DE, USA). The polymer samples were analyzed under a nitrogen gas purge of 10 mL/min at a heating rate of 10 °C min^−1^ and a temperature ranging from 25 to 700 °C.

The FT-IR was performed with a FT/IR 4600 spectrometer (JASCO). The polymer samples were heated at 200 °C for 18 h under a nitrogen gas atmosphere. After natural cooling, the spectra were recorded in transmittance mode within the range from 4000 cm^−1^ to 400 cm^−1^ with a resolution of 4 cm^−1^ and 16 scans were co-added.

## 3. Results and Discussion

### 3.1. Synthesis of Poly(IA-co-10-HHIA)s

**Figure 2:**
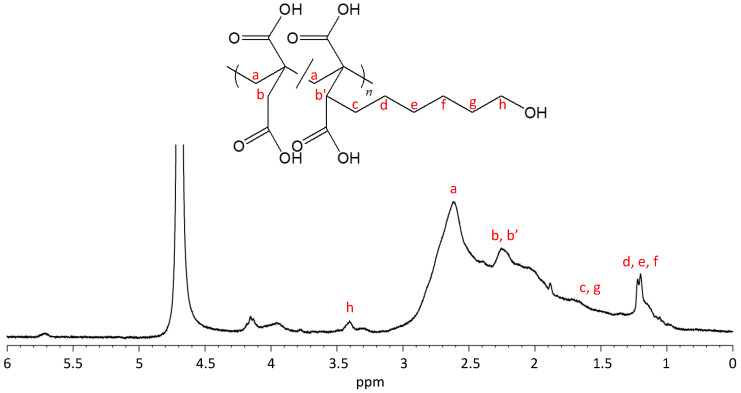
^1^H NMR spectrum of poly(IA-*co*-10-HHIA) synthesized with an IA/10-HHIA monomer ratio of 100/100 μmol (code 3). The letters indicate the positions of protons and their corresponding signals.

**Figure 3:**
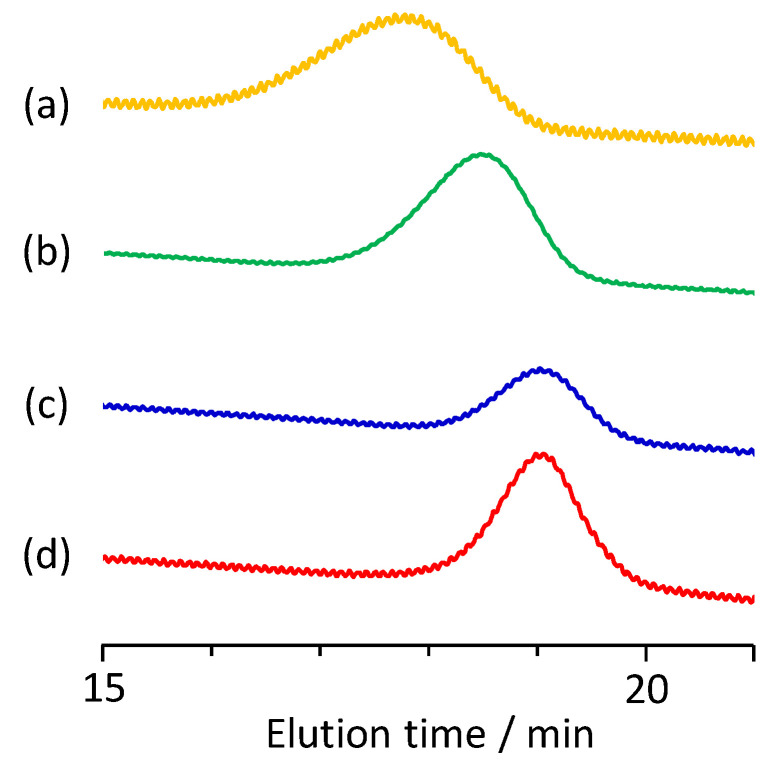
Gel permeation chromatography (GPC) traces of poly(IA-*co*-10-HHIA)s. (**a**) Code 1, (**b**) code 2, (**c**) code 3, (**d**) code 4.

According to reported homo-polymerizations of IA [29,30,31,32,33,34], the poly(IA-*co*-10-HHIA) copolymers were synthesized by the free radical polymerization of IA and 10-HHIA in water with KPS as an initiator. Figure 2 shows a representative ^1^H NMR spectrum of the poly(IA-*co*-10-HHIA) copolymer. The methylene signal at 2.7 ppm represents the polymer backbone. The signals at 2.2–2.3 ppm correspond to methine and methylene protons of IA and 10-HHIA. The signals at 1.3–1.6 and 3.4 ppm correspond to the methylene protons of the hexyl group of 10-HHIA. These results indicated that novel biobased polyvinyls could be synthesized using the naturally occurring IA derivative as a monomer. The reproducibility of the copolymer structure was confirmed because similar NMR spectra were obtained after the same experiment several times (data not shown). Figure 3 shows GPC curves of poly(IA-*co*-10-HHIA)s synthesized with different monomer feed ratios. The GPC curves show that all synthesized copolymers have a single peak, indicating that two distinct molecular weight species were absent in the samples analyzed.

Table 1 summarizes the polymerization conversion, the monomer compositions, the copolymer yields, the number-average molecular weight (*M*n), and the molecular weight distribution (MWD) of the molecular weight distribution of the copolymers with weight-average molecular weight (*M*w). The polymerization conversion, yield, *M*w, and *M*n of the synthesized poly(IA-*co*-10-HHIA)s decreased by increasing the feed ratio of 10-HHIA. Interestingly, all obtained copolymers have, despite the free radical polymerization, relatively low MWDs (1.10–1.29). A related paper reported that the homo-polymerization of IA in water with KPS required a relatively long reacting period (48 h) and the resulting poly(IA) showed low MWDs (1.12–1.14) [34]. Our results with 10-HHIA are in good agreement with results reported so far.

In summary, we demonstrated that 10-HHIA can be polymerized by free radical polymerization in the same way as IA, resulting in novel renewable polyvinyls, but the degree of polymerization of 10-HHIA is lower than that of IA; this may be due to the steric effect of the hydroxyhexyl group of 10-HHIA.

### 3.2. Thermal Characterization of Poly(IA-co-10-HHIA)s

**Figure 4:**
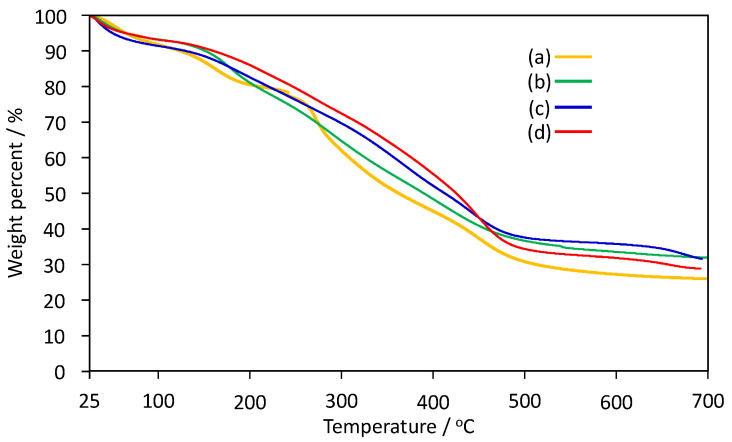
TGA thermograms of poly(IA-*co*-10-HHIA)s. (**a**) Code 1, (**b**) code 2, (**c**) code 3, (**d**) code 4.

To characterize the thermal properties of poly(IA-*co*-10-HHIA)s, TGA and FT-IR were conducted. Figure 4 shows TGA thermograms of poly(IA-*co*-10-HHIA)s from 25 to 700 °C. The TGA of all poly(IA-*co*-10-HHIA)s indicated a similar profile and they continuously decomposed as the temperature increased. Previous papers on TGA of poly(acrylic acid) and poly(IA) described similar results [31,35,36,37,38]. Krušić et al., have reported that the derivative thermogravimetry curve of poly(IA) has three maxima, at 185 °C, 315 °C, and 388 °C, assigned to the elimination of water and the formation of polyanhydride, followed by decarboxylation of anhydride groups and the breaking of the main polymer backbone [32,39]. Kayaman et al., have reported that poly(IA) showed a small weight loss at 100 °C implying the loss of moisture, and that poly(IA) had a significant weight loss at around 165 °C when TGA thermal decomposition was analyzed [37]. In addition, Ha et al., have reported that the carboxyl groups in poly(acrylic acid) were crosslinked by dehydration at 160 °C [40]. This suggested that dehydration occurred by linking the carboxyl groups of IA and 10-HHIA units by heating. Interestingly, the TGA results indicated that the poly(IA-*co*-10-HHIA)s continuously decomposed as temperature increased. This may be because the hydroxyhexyl group of 10-HHIA inhibited the formation of the linkage between the carboxyl groups of IA and 10-HHIA units. As the 10-HHIA content increased, the weight loss rate of poly(IA-*co*-10-HHIA)s was reduced. This may be due to the weight contribution of the carboxyl group in the copolymers decreased.

**Figure 5:**
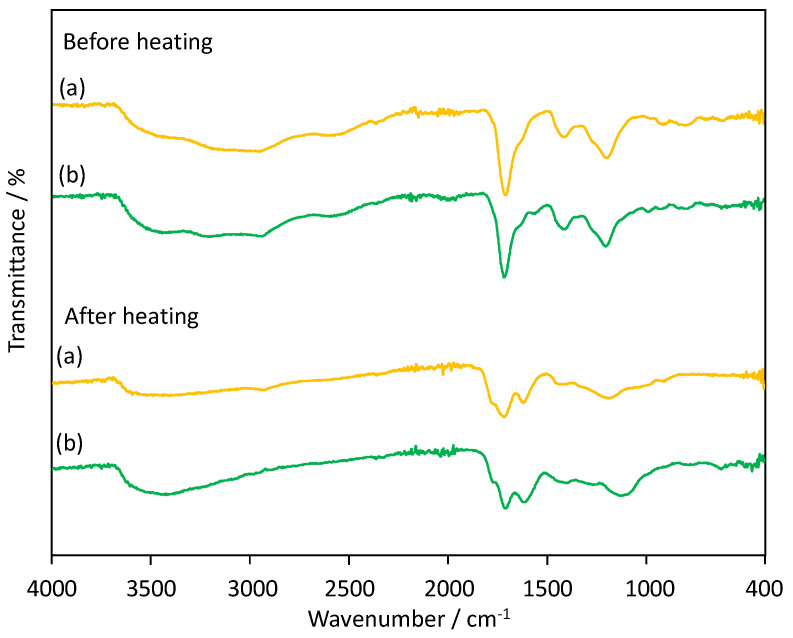
FT-IR spectra of (a) poly(IA) and (b) poly(IA-*co*-10-HHIA) synthesized with an IA/10-HHIA monomer ratio of 160/40 μmol (code 2) before and after heating at 200 °C for 18 h.

Figure 5 shows FT-IR spectra of poly(IA) and a representative poly(IA-*co*-10-HHIA) copolymer before and after heating at 200 °C for 18 h. The non-heated poly(IA) has the characteristic peaks at 2800–3400 cm^−1^, 1707 cm^−1^, 1639 cm^−1^, 1411 cm^−1^, 1193 cm^−1^, and 908 cm^−1^ assigned to a broad -OH stretching, the carboxylic acid (C=O) stretching, asymmetric C=O stretching of carboxylate anion, symmetric C=O stretching of carboxylate anion, C-O-H in-plane bending interactions, C-O stretching dimer, and O-H out-of-plane bending, respectively, according to the FT-IR spectrum of poly(IA) [37]. The non-heated poly(IA-*co*-10-HHIA) copolymer showed a similar profile. The poly(IA) and poly(IA-*co*-10-HHIA) copolymer heated at 200 °C for 18 h have a clear peak corresponding to the symmetric C=O stretching of carboxylate anion at 1610–1616 cm^−1^. This indicates that the heating promoted the formation of the hydrogen bonds between the carboxyl groups of IA and 10-HHIA. The intensity of the peak corresponding to the carboxylic acid (C=O) stretching at 1706–1715 cm^−1^ decreased after the heating. This suggests dissociation of the carboxyl groups of IA and 10-HHIA by the heating. It has been reported that heating poly(IA) results in the dehydration between carboxyl groups in the polymer and consequently the formation of poly(IA anhydride) [31,38]. These suggest that the formation of the hydrogen bonds between the carboxyl groups of IA and 10-HHIA in the polymer chains was promoted by heating and consequently the polymer dehydration occurred.

## 4. Conclusions

The present study reports the synthesis and thermal characterization of poly(IA-*co*-10-HHIA)s. The poly(IA-*co*-10-HHIA)s were synthesized by free radical polymerization with different monomer feed ratios (IA/10-HHIA: 200/0–0/200 μmol). The resulting polymers with higher 10-HHIA content showed a lower polymerization conversion, yield, *M*n, and *M*w of the synthesized copolymers. The copolymer MWDs were also relatively low (1.10–1.29). We related this to the steric effect of the hydroxyhexyl group of 10-HHIA. Thermal analyses suggested that heating promoted the formation of the hydrogen bonds between the carboxyl groups of IA and 10-HHIA in the polymer chains and then the polymer dehydration occurred. To the best of our knowledge, this is the first study that reports on the biobased polyvinyl synthesis using naturally occurring IA derivatives.

## Figures and Tables

**Table 1:** Synthesis of poly(IA-*co*-10-HHIA)s with different monomer feed ratios.

Code	IA/10-HHIA (μmol) ^1^	Conversion (%) ^2^		Composition (%) ^2^		Yield (%) ^3^	*M*n (g mol^−1^) ^4^	*M*w/*M*n ^4^
		IA	10-HHIA	IA	10-HHIA			
1	200/0	98	-	100	0	72	26,400	1.29
2	160/40	70	55	84	16	36	15,400	1.16
3	100/100	56	30	66	34	18	9870	1.08
4	0/200	-	16	0	100	11	9780	1.10

^1^ Monomer feed ratio. The volume of reaction solvent (water) was 0.4 mL. ^2^ Determined by ^1^H NMR. ^3^ Isolated yield. ^4^ Determined by GPC.
